# Health-related quality of life measured using the EQ-5D–5L: South Australian population norms

**DOI:** 10.1186/s12955-016-0537-0

**Published:** 2016-09-20

**Authors:** Nikki McCaffrey, Billingsley Kaambwa, David C. Currow, Julie Ratcliffe

**Affiliations:** 1 Flinders Health Economics Group, Flinders University, Repatriation General Hospital, Rm 55, Level 1, Block A, Repatriation General Hospital, Daws Road, Daw Park, SA 5041 Australia; 2 Palliative and Supportive Care, Bedford Park, Flinders University, Bedford Park, SA 5042 Australia

**Keywords:** EQ-5D, Population norms, Utility, Quality of life

## Abstract

**Background:**

Although a five level version of the widely-used EuroQol 5 dimensions (EQ-5D) instrument has been developed, population norms are not yet available for Australia to inform the future valuation of health in economic evaluations. The aim of this study was to estimate HrQOL normative values for the EQ-5D-5L preference-based measure in a large, randomly selected, community sample in South Australia.

**Methods:**

The EQ-5D-5L instrument was included in the 2013 South Australian Health Omnibus Survey, an interviewer-administered, face-to-face, cross-sectional survey. Respondents rated their level of impairment across dimensions (mobility, self-care, usual activities, pain/discomfort, and anxiety/depression) and global health rating on a visual analogue scale (EQ-VAS). Utility scores were derived using the newly-developed UK general population-based algorithm and relationships between utility and EQ-VAS scores and socio-demographic factors were also explored using multivariate regression analyses.

**Results:**

Ultimately, 2,908 adults participated in the survey (63.4 % participation rate). The mean utility and EQ-VAS scores were 0.91 (95 CI 0.90, 0.91) and 78.55 (95 % CI 77.95, 79.15), respectively. Almost half of respondents reported no problems across all dimensions (42.8 %), whereas only 7.2 % rated their health >90 on the EQ-VAS (100 = the best health you can imagine). Younger age, male gender, longer duration of education, higher annual household income, employment and marriage/de facto relationships were all independent, statistically significant predictors of better health status (*p* < 0.01) measured with the EQ-VAS. Only age and employment status were associated with higher utility scores, indicating fundamental differences between these measures of health status.

**Conclusions:**

This is the first Australian study to apply the EQ-5D-5L in a large, community sample. Overall, findings are consistent with EQ-5D-5L utility and VAS scores reported for other countries and indicate that the majority of South Australian adults report themselves in full health. When valuing health in Australian economic evaluations, the utility population norms can be used to estimate HrQOL. More generally, the EQ-VAS score may be a better measure of population health given the smaller ceiling effect and broader coverage of HrQOL dimensions. Further research is recommended to update EQ-5D-5L population norms using the Australian general population specific scoring algorithm once this becomes publically available.

## Background

Increasingly, economic evaluations are used to inform clinical, funding, public and health policy decisions [1–3]. Economic evaluations systematically compare the relative costs and benefits of competing courses of action, informing choices on how best to maximise benefits within budget-constrained funds [4]. One of the most commonly reported measures of benefit in economic evaluations is the quality adjusted life year (QALY) [5] which combines health-related quality of life (HrQOL) and length of life into a single index summary measure. Health-related quality of life is represented by quality weights (utilities) typically measured on a ‘0’ to ‘1’ scale where ‘0’ is defined as a health state equivalent to being dead and ‘1’ is full health. Generic, preference-based, multi-attribute utility instruments (MAUIs) such as the EuroQol 5 dimensions (EQ-5D) are the most popular mechanism for indirectly estimating these utilities [5]. Generic MAUIs have two main elements: a set of items with multiple response categories covering different dimensions of HrQOL (descriptive system); and an off-the-shelf scoring algorithm indicating the strength of preference for the health states defined by the instrument (quality weights). Typically, scoring algorithms are generated from large general population surveys to elicit values for a selection of health states (value set) defined by the descriptive system [5].

The EQ-5D was first developed in 1990 and is the world’s most widely applied generic MAUI [5]. The original descriptive system has five dimensions (mobility, self-care, usual activities, pain/discomfort, anxiety/depression), each with three response levels (EQ-5D-3L). A five response level version has been developed (EQ-5D-5L) in an effort to reduce the potential for ceiling effects and to address concerns about the sensitivity of the 3L version for detecting clinically important differences in HrQOL [6]. Emerging evidence suggests the newer 5-level version does have improved measurement properties including feasibility, ceiling effects, sensitivity and convergent validity and therefore may be more useful for measuring population-level health status [7–11]. A new off-the-shelf scoring algorithm estimated from a sample of the UK adult general population is now available for this version [12].

Health-related quality of life is also often used as a measure of population health status to inform public health and health care policy [13]. Population-wide studies of HrQOL facilitate surveillance of health status over time, identify groups at risk of poorer HrQOL, enable assessment of the burden of different diseases on HrQOL and capture the relationships between socio-demographic characteristics and health status [13–15]. Consideration of the EQ-5D evidence to date suggests younger age, male gender and longer duration of education are associated with better health status when measured using this measure [7, 11, 16–21]. Previous population-wide studies using the 3L version suggest household income, employment and marital status may also be associated with HrQOL [14, 22–35].

It is highly likely the 5L version will replace the 3L version as the instrument of choice in future health economic evaluations and population-wide studies. Although EQ-5D-5L population norms and relationships with socio-demographic characteristics have already been reported for Canada [16], Germany [17]; Italy [18], Japan [19], Poland [20], Spain [11], Uruguay [21], and the UK [7], none are yet available for Australia.

### Objectives

The objectives of this study were to:

1. Estimate population norms for the EQ-5D-5L for South Australia using a large, randomly selected, community sample and the new UK scoring algorithm to inform economic evaluations; and
2. Examine the relationships between socio-demographic factors and HrQOL measured using EQ-5D-5L utility and VAS scores.

## Methods

The STROBE (Strengthening the Reporting of Observational Studies in Epidemiology) guidelines were followed during the preparation of this manuscript [36].

### Study design

The South Australian Health Omnibus Survey (HOS) is an annual, interviewer-administered, face-to-face, cross-sectional observational study of a clustered area sample of households identified using a multistage, systematic, randomised approach [37].

### Data collection

The EQ-5D-5L instrument was included in the 2013 South Australian Health Omnibus Survey (HOS). The HOS samples included 5,200 households randomly selected from Statistical Areas Level 1, from metropolitan Adelaide area and country towns with a population of 1,000 people or more. The HOS contains questions submitted by researchers on a user-pays basis. Typically, about 200 questions on health and social-related issues are included, with interviews conducted in the respondent’s home lasting 60–90 min [38]. One interview was conducted per household with verbally consented participants over the age of 15 years who most recently had a birthday. Interviews were conducted between 3^rd^ September and 31^st^ December 2013 and de-identified socio-demographic data and EQ-5D-5L responses were collected.

### EQ-5D-5L

Each dimension in the EQ-5D-5L has five response levels: no problems (Level 1); slight; moderate; severe; and extreme problems (Level 5). There are 3,125 possible health states defined by combining one level from each dimension, ranging from 11111 (full health) to 55555 (worst health) [6]. EQ-5D-5L health states are converted into a single index ‘utility’ score using a scoring algorithm based on public preferences. In this study, the UK value set and scoring algorithm were used to calculate utility scores as an Australian scoring algorithm is not yet available for the 5L. The UK algorithm was estimated using a hybrid model of preference data collected using a time-trade off (TTO) and discrete choice experiment (DCE) [12, 39] and potential values from this algorithm ranged from -0.281 to 1, where values lower than 0 represent states considered to be worse than death [12]. The instrument also includes a visual analogue scale (EQ-VAS) which provides a single global rating of self-perceived health and is scored on a 0 to 100 mm scale representing “the worst…” and “the best health you can imagine”, respectively.

### Socio-demographic characteristics

Socio-demographic characteristics collected in the HOS survey included age, area of residence, country of birth, educational attainment, employment status, gender, gross annual household income; and marital status.

### Data analysis

Analyses were performed using SPSS for Windows version 22.0 (SPSS, Inc., Chicago, IL) and Stata version 13.1. [40]. The HOS data were weighted by the inverse of the respondent’s probability of selection for the survey, the response rates in metropolitan and country regions, and re-weighted to benchmarks from the 2011 Census to provide a demographic description of the South Australian population by age groups, gender and geographic profile [41].

Descriptive summary statistics were estimated for socio-demographic variables, the EQ-5D-5L dimensions, utility scores, EQ-VAS scores and the top 20 most frequently reported EQ-5D-5L health states [7, 42, 43]. A priori hypothesised correlations (presented in brackets), based on a review of the literature, between utility scores and EQ-VAS scores (moderate, positive) and age (strong, negative) [22, 44–46] were evaluated with Spearman’s correlation coefficients. Correlations of <0.30 were considered weak, 0.30–0.50 moderate and >0.50 strong [47] and were judged statistically significant using a Bonferroni adjusted alpha level of 0.017 (0.05/3) [48]. Socio-demographic characteristics were categorised as shown in Table 1. As the EQ-5D-5L utility scores were non-normally distributed (Kolmogorov-Smirnov test, *p* < 0.05), differences between socio-demographic sub-groups were assessed using the non-parametric Mann Whitney *U* test (two groups) and Kruskal-Wallis one way analysis of variance (multiple groups) at the 0.00625 alpha level, following adjustment for multiple testing (0.05/8) [49, 50].

**Table 1:** Socio-demographic characteristics of the South Australian Health Omnibus Survey sample

Sample	Total	Males	Females
	*N* = 2,908	*n* = 1,422	*n* = 1,486
Age, mean (SD)	46.3 (18.9)	45.7 (18.6)	47.0 (19.1)
Age category, years, %			
15–24	15.9	16.7	15.2
25–34	15.4	15.8	15.1
35–44	16.5	16.8	16.3
45–54	17.2	17.4	17.0
55–64	15.2	15.3	15.2
65–74	11.9	10.8	13.0
75+	7.8	7.3	8.2
Gender, % female	51.1		
Education attainment, %			
Left school at ≤15 years of age	9.1	6.6	11.5
Left school >15 years of age	21.5	19.0	24.0
Trade, apprenticeship, certificate, diploma	36.5	42.5	30.7
Degree or higher	22.4	21.1	23.6
Still studying	10.2	10.2	10.1
Not stated	0.3	0.5	0.1
Area of residence, %			
Metropolitan	74.4	75.5	73.6
Regional	25.6	24.5	26.4
Country of birth, %			
Australia	73.5	74.1	72.9
Europe	15.8	15.2	16.4
Asia	7.1	7.7	6.4
Other	3.5	2.7	4.2
Not stated	0.1	0.1	0.1
Marital status, %			
Married/De Facto	61.5	63.4	59.9
Never Married	24.7	27.7	21.8
Separated/divorced	9.1	6.6	11.4
Widowed	4.6	2.2	6.8
Not stated	0.1	0.1	0.1
Employment status, %			
Full or part time	55.6	62.8	48.6
Unemployed	3.3	3.2	3.4
Not in the labour force	41.1	33.9	47.8
Home Duties	5.5	0.4	10.4
Retired	20.4	18.0	22.7
Student	9.5	9.2	9.8
Not working due to work-related injury or disability	4.1	4.7	3.4
Other	1.6	1.6	1.5
Not stated	0.1	0.1	0.1
Annual household income, %			
Up to $20,000	6.0	4.1	7.9
$20,001–$40,000	12.8	11.2	14.4
$40,001–$80,000	19.3	19.7	19.0
$80,001–$120,000	15.1	16.2	14.0
$120,001+	20.1	23.8	16.6
Not stated	26.7	25.0	28.2

*SD* standard deviation

The relationships between socio-demographic variables and EQ-5D-5L utility scores were explored using a generalized linear model (GLM) with a Poisson distribution and a log link [51, 52]. This model controls for skewness and heteroscedasticity and approximates the distribution of the data based on the modified Park test [53, 54]. The EQ-5D-5L *disutility* score (1-utility score) was entered as the dependent variable as positive values are required for the specified regression model [52]. Explanatory variables were limited to those included in the HOS survey. Based on a literature review of previously reported EQ-5D-5L population norms, older respondents, females and those with a shorter duration of education were expected a priori to report higher disutility scores (poorer health status) and were entered first in the GLM (Model 1) [7, 11, 16–21]. Subsequently, a series of exploratory multivariate regression analyses were conducted for individual variables which were statistically significant in the bivariate analysis, controlling for age, gender and education. Factors which were statistically significant in these exploratory analyses were then included in the final model to assess whether these variables independently predicted HrQOL (Model 2). Disutility scores were hypothesised to increase with lower gross annual household income; unemployed persons were expected to have higher disutility scores than employed persons; and widowed, separated and divorced people were anticipated to have higher disutility scores than married people [14, 22–35]. The relationships between socio-demographic variables and EQ-VAS scores were similarly explored using a GLM with a Poisson distribution and a log link based on the modified Park test [53, 54].

## Results

### Participants

**Fig. 1:**
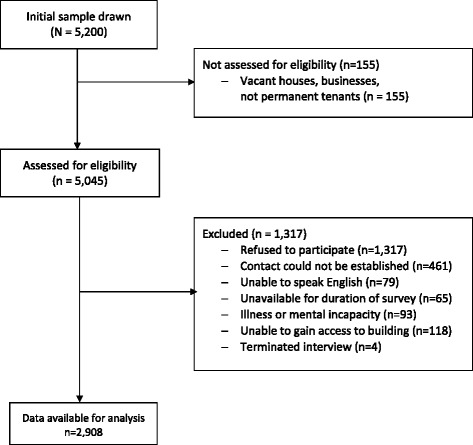
STROBE participant flow diagram

In total, 2,908 individuals participated in the 2013 Heath Omnibus Survey, achieving a participation rate of 63.4 % (Fig. 1). The socio-demographic characteristics of the respondents are summarised in Table 1.

### Main results

**Fig. 2:**
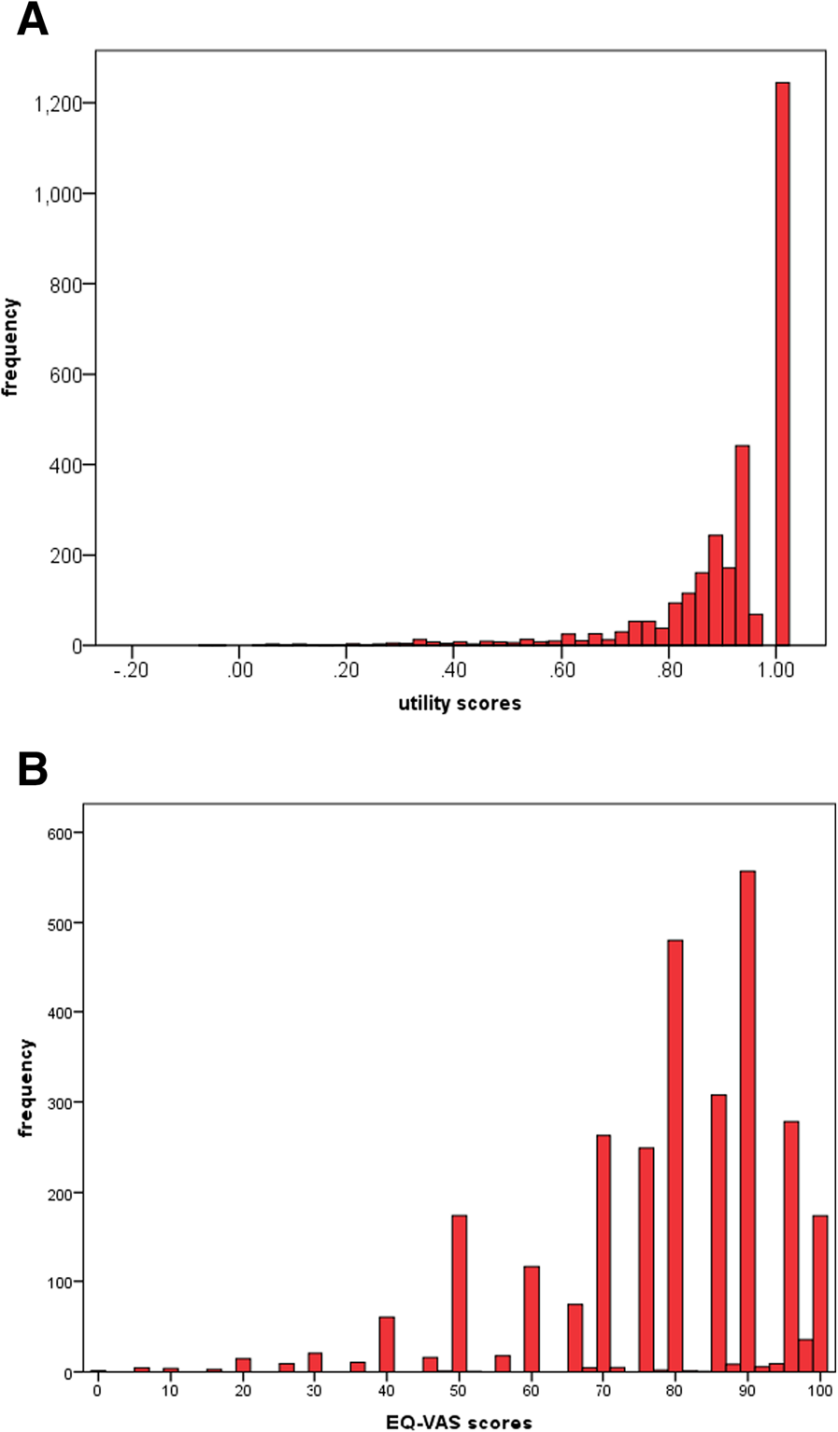
**a** Distribution of EQ-5D-5L scores (*N* = 2,908); **b** Distribution of EQ-VAS scores (*N* = 2,908)

The EQ-5D-5L utility scores were heavily left-skewed with a clustering at 1.00, ‘full health’ (Fig. 2a). The EQ-VAS scores were also left-skewed, although to a lesser extent, and responses clustered predominantly around 80 and 90 on the 100 mm scale (Fig. 2b). The frequencies of item responses for each EQ-5D-5L dimension are presented in Table 2. As expected in a community-based general population sample, a substantial proportion of respondents reported no problems on any of the five dimensions (42.8 %). The most prevalent problems were reported for pain and discomfort (44.4 %).

**Table 2:** Frequencies of item responses in each EQ-5D-5L dimension by age and gender (%)

	Age category, years							
Dimension	Total *N* = 2,908	15–24 *n* = 464	25–34 *n* = 449	35–44 *n* = 480	45–54 *n* = 499	55–64 *n* = 443	65–74 *n* = 346	75+ *n* = 226
Mobility								
No problems	74.3	93.1	89.3	82.9	73.4	63.9	54.6	40.3
Slight	14.9	4.1	8.0	10.8	15.0	22.6	24.9	29.2
Moderate	7.9	2.4	1.6	4.4	8.4	10.6	14.2	23.0
Severe	2.6	0	0.9	1.7	3.0	2.9	6.1	5.8
Extreme	0.3	0.4	0.2	0.2	0.2	0	0.3	1.8
Self-care								
No problems	95.4	98.5	99.3	96.9	95.4	94.4	90.8	87.1
Slight	3.4	1.3	0.2	2.5	3.8	3.8	6.9	8.9
Moderate	0.9	0	0.4	0.6	0.2	1.1	2.0	3.1
Severe	0.3	0	0	0	0.6	0.5	0	0.9
Extreme	0.1	0.2	0	0	0	0.2	0.3	0
Usual activities								
No problems	82.7	94.4	94.0	87.8	78.8	78.6	72.3	56.6
Slight	10.2	5.0	4.2	8.3	12.2	12.8	13.6	22.1
Moderate	5.0	0.4	0.9	2.9	6.0	6.1	9.8	15.5
Severe	1.6	0	0.4	0.8	2.6	1.8	2.9	4.4
Extreme	0.5	0.2	0.4	0.2	0.4	0.7	1.4	1.3
Pain/Discomfort								
No pain	55.6	80.8	70.8	62.7	46.9	41.1	40.2	29.6
Slight	29.2	16.8	23.6	26.0	34.5	36.8	34.7	38.1
Moderate	11.9	2.4	4.9	7.7	13.8	17.8	20.2	26.1
Severe	2.7	0	0.4	2.7	4.0	3.4	4.3	5.8
Extreme	0.6	0	0.2	0.8	0.8	0.9	0.6	0.4
Anxiety/Depression								
No problems	75.3	77.5	81.3	73.6	70.1	75.7	76.0	73.0
Slight	16.3	14.7	11.4	16.6	19.4	16.7	16.2	20.8
Moderate	6.2	5.8	5.6	7.3	6.4	5.9	6.9	4.9
Severe	1.6	1.3	1.8	1.7	3.0	0.9	0.6	1.3
Extreme	0.6	0.6	0	0.8	1.0	0.9	0.3	0

The mean utility score was 0.91 (SD 0.14; 95 % CI 0.90, 0.91) with values ranging from -0.06 to 1 (Table 3) and the mean EQ-VAS score was 78.55 (SD 16.57; 95 % CI 77.95, 79.15) (Table 4). Ten out of the 3,125 possible health states represented the majority of the sample (76.2 %) (Table 5). Individuals rating themselves as ‘11111’, assigned a mean score of 85.44 to their health state on the EQ-VAS. Overall, there was a moderate, positive statistically significant correlation between utility and EQ-VAS scores (rho = 0.46, *p* < 0.001) and a moderate, negative, statistically significant correlation between utility scores and age (rho = -0.31, *p* < 0.001).

**Table 3:** Mean EQ-5D-5L utility scores by socio-demographic characteristics (*N* = 2,908)

	Total		Males		Females	
Variable	*n*	mean (SD)	*n*	mean (SD)	*n*	mean (SD)
All	2908	0.91 (0.14)	1422	0.92 (0.13)	1486	0.90 (0.14)
			*Z* = -3.42, *p* = 0.001			
Age category, years						
15–24	464	0.96 (0.08)	238	0.96 (0.07)	226	0.95 (0.08)
25–34	449	0.95 (0.10)	225	0.95 (0.10)	224	0.95 (0.11)
35–44	480	0.92 (0.13)	238	0.93 (0.12)	241	0.91 (0.13)
45–54	499	0.89 (0.16)	247	0.90 (0.16)	253	0.87 (0.16)
55–64	443	0.89 (0.15)	217	0.90 (0.14)	226	0.88 (0.15)
65–74	346	0.87 (0.16)	153	0.87 (0.16)	193	0.87 (0.16)
75+	227	0.83 (0.16)	104	0.85 (0.16)	122	0.82 (0.15)
Total	2908	*X* ^2^ = 282, *p* < 0.001				
Educational attainment						
Up to secondary	1187	0.90 (0.15)	510	0.91 (0.16)	677	0.89 (0.15)
Trade, apprenticeship, certificate, diploma	1061	0.90 (0.14)	605	0.91 (0.12)	456	0.89 (0.15)
Degree or higher	651	0.93 (0.12)	301	0.93 (0.12)	351	0.93 (0.12)
Total	2900	*X* ^2^ = 15.0, *p* = 0.001				
Area of residence						
Metropolitan	2163	0.91 (0.14)	1047	0.92 (0.13)	1114	0.90 (0.14)
Regional	745	0.90 (0.15)	373	0.91 (0.14)	372	0.90 (0.15)
Total	2908	*Z* = -0.21, *p* = 0.83				
Country of birth						
Australia	2138	0.91 (0.14)	1054	0.91 (0.14)	1082	0.90 (0.14)
European	461	0.88 (0.16)	217	0.90 (0.14)	244	0.86 (0.17)
Asian	206	0.97 (0.07)	110	0.97 (0.06)	96	0.96 (0.08)
Other	101	0.93 (0.12)	39	0.93 (0.15)	62	0.94 (0.10)
Total	2906	*X* ^2^ = 75.6, *p* < 0.001				
Marital status						
Married/De Facto	1791	0.91 (0.14)	900	0.92 (0.13)	890	0.90 (0.14)
Never Married	718	0.93 (0.13)	394	0.93 (0.14)	325	0.93 (0.12)
Separated/Divorced	264	0.87 (0.17)	94	0.88 (0.17)	169	0.86 (0.16)
Widowed	133	0.84 (0.16)	31	0.87 (0.15)	100	0.83 (0.16)
Total	2906	*X* ^2^ = 92.8, *p* < 0.001				
Employment status						
Full time or part-time	1616	0.94 (0.09)	891	0.95 (0.08)	723	0.93 (0.10)
Home Duties	161	0.90 (0.14)	6	0.96 (0.04)	155	0.90 (0.14)
Unemployed	96	0.88 (0.15)	45	0.90 (0.15)	51	0.87 (0.15)
Retired	593	0.86 (0.15)	256	0.87 (0.15)	337	0.86 (0.15)
Student	275	0.95 (0.07)	130	0.96 (0.08)	145	0.95 (0.07)
Not working due to work-related injury or disability	118	0.61 (0.26)	67	0.62 (0.26)	51	0.60 (0.26)
Other	45	0.88 (0.16)	23	0.96 (0.06)	23	0.81 (0.19)
Total	2904	*X* ^2^ = 407, *p* < 0.001				
Annual household income						
Up to $20,000	176	0.83 (0.19)	59	0.83 (0.20)	117	0.82 (0.19)
$20,001-$40,000	372	0.84 (0.17)	159	0.84 (0.18)	214	0.85 (0.17)
$40,001-$80,000	562	0.91 (0.14)	280	0.92 (0.13)	282	0.90 (0.15)
$80,001-$120,000	438	0.93 (0.10)	230	0.94 (0.10)	208	0.93 (0.11)
$120,001+	584	0.94 (0.10)	338	0.95 (0.08)	246	0.93 (0.11)
Not Stated	776	0.91 (0.14)	354	0.92 (0.15)	417	0.91 (0.14)
Total	2908	*X* ^2^ = 156, *p* < 0.001				

*SD* standard deviation; comparisons of the EQ-5D-5L sum score distributions by gender and area of residence were analysed using the Mann-Whitney *U* test. All other differences amongst groups were analysed with the Kruskal-Wallis test

**Table 4:** Mean EQ-VAS scores by age and gender (*N* = 2,908)

	Total		Males		Females	
Variable	*n*	mean (SD)	*n*	mean (SD)	*n*	mean (SD)
All	2905	78.55 (16.57)	1422	78.89 (15.71)	1483	78.23 (17.35)
			*Z* = -1.29, *p* = 0.20			
Age category, years						
15–24	464	82.00 (14.06)	238	81.75 (13.02)	226	82.26 (15.11)
25–34	449	80.84 (14.58)	225	81.51 (14.56)	224	80.16 (14.60)
35–44	480	78.50 (16.65)	238	79.05 (15.03)	241	77.96 (18.13)
45–54	499	75.64 (18.00)	247	76.08 (16.94)	253	75.20 (18.96)
55–64	443	78.92 (16.84)	217	79.01 (16.02)	225	78.83 (17.63)
65–74	346	78.56 (17.19)	153	78.21 (17.04)	193	78.83 (17.34)
75+	225	72.68 (17.66)	104	73.66 (17.38)	121	71.83 (17.93)
Total	2906	*X* ^2 =^ 58.8, *p* < 0.001				

*SD* standard deviation, comparisons of the EQ-VAS score distributions by gender was analysed using the Mann-Whitney *U* test. Differences amongst age groups were analysed with the Kruskal-Wallis test

**Table 5:** Most frequently reported EQ-5D-5L health states with mean utility scores and EQ-VAS values (*N* = 2,908)

Health state^a^	*n*	%	Cumulative %	Mean utility	Mean EQ-VAS (SD)
11111	1245	42.8	42.8	1.00	85.44 (11.75)
11121	356	12.2	55.0	0.94	81.32 (13.02)
11112	162	5.6	60.6	0.92	79.20 (13.33)
21121	116	4.0	64.6	0.89	80.71 (12.75)
11122	93	3.2	67.8	0.87	77.72 (14.08)
11131	69	2.4	70.2	0.93	74.67 (14.67)
21111	53	1.8	72.0	0.95	83.56 (8.70)
11113	52	1.8	73.8	0.90	71.99 (15.4)
21221	41	1.4	75.2	0.84	76.89 (14.90)
21122	30	1.0	76.2	0.82	74.41 (12.56)
11221	24	0.8	77.0	0.89	78.70 (11.68)
21131	24	0.8	77.8	0.88	76.69 (13.63)
11123	23	0.8	78.6	0.84	70.91 (15.28)
11132	19	0.6	79.2	0.85	71.58 (18.01)
21222	18	0.6	79.8	0.77	64.75 (16.82)
31231	17	0.6	80.4	0.82	66.86 (15.00)
31221	16	0.6	81.0	0.83	70.92 (17.31)
21231	16	0.6	81.6	0.83	63.27 (14.33)
11211	15	0.5	82.1	0.95	73.14 (17.48)
31121	15	0.5	82.6	0.88	77.07 (15.60)

^a^digits represent response levels (1–5) for the five dimensions (mobility, self-care, usual activities, pain/discomfort, anxiety/depression); *EQ-VAS* visual analogue scale

The mean EQ-5D-5L utility scores by socio-demographic variables and mean EQ-VAS scores by age and gender are summarised in Tables 3 and 4 respectively. Overall, men had statistically significantly higher utility scores than women (mean 0.92 (SD 0.13) versus 0.90 (SD 0.13); *Z* = -3.42, *p* < 0.01), although EQ-VAS scores were similar. Lower utility scores were reported with advancing age categories (chi-squared = 282, *p* < 0.01) and this relationship was somewhat U-shaped when EQ-VAS scores were considered. In the bivariate analyses, there were statistically significant differences in utility scores for the whole sample in terms of different marital statuses, educational attainment, employment statuses, country of birth and annual household income categories but not for areas of residence. After controlling for age, gender and duration of education, individual variables which remained significant in the exploratory multivariate regression analyses included employment, household income and marital status (data not shown). In the final model, advancing age was significantly associated with a negative impact on health status and disutility was higher for those unemployed or not working due to work-related injury or disability (Table 6). In addition, male gender, longer duration of education, higher annual household income and marriage or de facto relationships were also independent, statistically significant predictors of better health in the EQ-VAS multivariate regression analysis (Table 6).

**Table 6:** Multivariate Poisson regression analyses of EQ-5D-5L disutility scores, EQ-VAS scores and socio-demographic variables

Variable^a^	Disutility scores						EQ-VAS scores					
	Model 1			Model 2			Model 1			Model 2		
	*B* ^*b*^	95 % CI	*p*-value	*B* ^*b*^	95 % CI	*p*-value	*B* ^*b*^	95 % CI	*p*-value	*B* ^*b*^	95 % CI	*p*-value
***Control variables***												
Gender (positive)												
Male^c^												
Female	0.18	−0.06, 0.43	0.14	0.19	−0.05, 0.44	0.13	**0.01**	**0.01, 0.02**	**<0.01**	**0.02**	**0.01, 0.02**	**<0.01**
Age category, years (positive)												
15–29^c^												
30–49	**0.68**	**0.25, 1.11**	**<0.01**	**0.62**	**0.13, 1.10**	**0.01**	**−0.06**	**−0.07,−0.05**	**<0.01**	**−0.06**	**−0.07,−0.05**	**<0.01**
50–69	**0.98**	**0.58, 1.40**	**<0.01**	**0.85**	**0.34, 1.36**	**<0.01**	**−0.05**	**−0.06, −0.03**	**<0.01**	**−0.05**	**−0.06, −0.03**	**<0.01**
70+	**1.28**	**0.84, 1.72**	**<0.01**	**1.25**	**0.58, 1.92**	**<0.01**	**−0.63**	**−0.08, −0.05**	**<0.01**	**−0.10**	**−0.02, −0.07**	**<0.01**
Education attainment (negative)												
Up to secondary^c^												
Trade, apprenticeship, certificate, diploma	−0.05	−0.32, 0.22	0.71	0.07	−0.21, 0.34	0.63	**0.02**	**0.01, 0.03**	**<0.01**	**0.01**	**0.00, 0.02**	**0.04**
Degree or higher	−0.32	−0.67, 0.03	0.07	−0.07	−0.43, 0.30	0.72	**0.07**	**0.06, 0.08**	**<0.01**	**0.05**	**0.03, 0.06**	**<0.01**
***Exploratory variables***												
Annual household income (negative)												
Up to $20,000^c^												
$20,001–$60,000				−0.01	−0.44, 0.43	0.98				**0.02**	**0.00, 0.05**	**0.02**
$60,001–$100,000				−0.25	−0.80, 0.29	0.37				**0.07**	**0.05, 0.09**	**<0.01**
$100,001+				−0.33	−0.88, 0.22	0.24				**0.04**	**0.01, 0.06**	**<0.01**
Not stated				−0.17	−0.62, 0.29	0.48				**0.02**	**0.00, 0.04**	**0.04**
Employment status (positive)												
Full or part time, student, home duties^c^												
Unemployed				**0.67**	**0.04, 1.29**	**0.04**				**−0.09**	**−0.11, −0.07**	**<0.01**
Retired				0.14	−0.30, 0.58	0.54				**0.03**	**0.02, 0.05**	**<0.01**
Other^d^				**1.41**	**1.06, 1.77**	**<0.01**				**−0.31**	**−0.33, −0.29**	**<0.01**
Marital status (positive)												
Married/De Facto^c^												
Never Married				0.09	−0.32, 0.50	0.68				**−0.22**	**−0.04, −0.01**	**<0.01**
Separated/Divorced				0.57	−0.33, 0.44	0.77				**−0.06**	**−0.07, −0.04**	**<0.01**
Widowed				0.51	−0.45, 0.55	0.84				**−0.05**	**−0.08, −0.03**	**<0.01**
Constant	**−3.17**	**−3.57, −2.78**	**<0.01**	**−3.29**	**−3.99, −2.59**	**<0.01**	**4.38**	**4.37, 4.39**	**<0.01**	**4.39**	**4.37, 4.42**	**<0.01**
AIC	0.55			0.53			12.97			12.53		
BIC	−22714			−22703			−3367			−4589		

^a^The direction of a priori hypothesied associations relative to the reference group are presented in brackets; ^b^coefficient; ^c^reference group; ^d^other, not working due to work-related injury or disability; *AIC* Akaike information criteria, *BIC* Bayesian information criteria

Significant results at the *p* < 0.05 level are shown in bold

## Discussion

This is the first study to report EQ-5D-5L data for a large, randomly selected, community-based general population sample in Australia calculated using a scoring algorithm specifically developed for the five level version. These results provide important insights into the HrQOL of the South Australian population. Overall, the mean utility score (0.91) was similar to those recently reported using the EQ-5D-5L instrument for populations in Italy (0.92) [18], Germany (0.92) [17] and Poland (0.89) [20] and higher than the previously reported Australian value (0.87) measured using the 3L version in a representative adult sample in Queensland, Australia [22]. The difference in the Australian values could be due to variation in the populations sampled, the value sets employed [55] or versions of the EQ-5D administered.

The prevalence of the most frequently reported EQ-5D-5L health states were similar to those reported recently in a large survey in the UK (*N* = 996) [7] except for the health state representing slight problems in mobility and pain/discomfort and no problems in self-care, usual activities and anxiety/depression (health state 21121), which was more prevalent in the South Australian sample (4.0 % versus 2.1 %). This South Australian community-based population reported greater problems with pain and discomfort compared with other dimensions, similar to previously reported EQ-5D-5L population norms from other countries [7, 11, 17, 18, 20].

Primarily, the findings indicate that, in general, South Australians report high HrQOL according to the EQ-5D-5L classification with 42.8 % of respondents reporting no problems (11111) consistent with other EQ-5D-5L data generated from large general population samples in developed countries such as Germany (47.5 %) and the UK (47.6 %) [7, 17]. Although these data suggest a reduced ceiling effect for the 5L as a lower proportion of respondents reported no problems for each individual dimension compared with 3L general population sample data in Queensland, Australia [22] there remains a considerable proportion of respondents who report full health. Only 7.2 of respondents reported their health above 90 on the EQ-VAS and 4.7 % “the best health you can imagine”, suggesting ceiling effects persist with the utility scores. Consequently, the EQ-VAS scores may be a more appropriate measure of population global health rating [7, 56].

Consistent with findings from other countries [7, 25, 26, 32–34], collectively, the multivariate regression analyses suggested respondents who were younger, male, had higher levels of education or household income, were employed or married/de facto, were more likely to have a better health status. However, only age and employment status were independently associated with health status when utility, rather than the EQ-VAS scores, were considered, perhaps indicating fundamental differences between these measures. Further, despite mean utility and EQ-VAS scores declining with age, as anticipated a priori [7, 17, 23, 32, 35, 57], this association was more U-shaped for the latter scores, with a nadir during 45–54 years. This pattern is similar to the relationship between wellbeing and age [57] and indicates the EQ-VAS may cover broader dimensions of HrQOL than those included in the EQ-5D-5L descriptive system [56].

The EQ-VAS provides a comprehensive rating of overall health at the individual and population-level [56] and has been used to routinely monitor patients’ self-reported health in the National Health Service in England, monitor population health over time and estimate the burden of different diseases [55]. Purported advantages of the EQ-VAS include robust psychometric properties and simplicity and ease of use, although recent evidence suggests 55 % of respondents do not strictly adhere to the scoring instructions, presenting challenges for data analysis [5, 56]. Further, respondents’ interpretation of the ends of the scale may be different, potentially limiting comparability of EQ-VAS scores. Despite these limitations, the EQ-VAS scores are not subject to artificial differences introduced by the choice of different value sets [55, 58] and perhaps more accurately capture a global health rating from the individual’s perspective [56]. Ultimately, when considering the valuation of health in economic evaluations, regulatory reimbursement guidelines typically recommend using preference elicitation techniques such as standard gamble or time-trade off [59–61], whereas the EQ-VAS is choice-less [5].

This study presents findings from a large representative survey sample from a single state and likely reflects Australian norms but may not be generalizable to other countries. Compared with the rest of Australia, South Australians are slightly older (16.1 % ≥65 years of age versus 14.0 %), less educated (people aged ≥15 years who have completed Year 12 44.8 % versus 49.2 %; 15–64 year olds participating in vocational education 10.9 % versus 12.0 %; 25–64 years old with a Bachelor degree or above 22.9 % versus 27.9 %), have a lower mean weekly household income ($798 versus $848), and a higher rate of unemployment (5.6 % versus 5.2 %) [62]. Consequently, the population norms reported for the EQ-5D-5L for South Australia may be lower than those for the wider Australian general population. Further, individuals who live in remote areas and culturally and linguistically diverse populations were under-represented in the survey.

A UK, rather than Australian, value set was used to calculate utility scores as the latter is not yet available. Previous evidence comparing EQ-5D-3L population norms in Queensland, Australia [22] estimated using value sets from Australia, the UK and USA, suggests the UK value set provides relatively comparable valuations, although higher health states have been reported elsewhere when using the Australian algorithm [63]. Generally, guidelines recommend using preference weights specific to the jurisdiction of interest [59, 64] as empirical evidence suggests population values may differ for health states across countries, possibly due to cultural differences [31, 63, 65]. Previously, Norman et al [66] recently published a pilot scoring algorithm to generate Australian general population-specific utilities and an Australian general population-specific scoring algorithm for the EQ-5D-5L is currently in development^1^.

The utility and EQ-VAS results provide clinicians, funders and policy-makers with an alternative set of population norms to monitor policy changes and inform future public health and health care investment decisions. In the Australian setting, when the EQ-5D is the instrument of choice for valuing health in economic evaluations, population norms for South Australia or Queensland [22] can be used to estimate health-related quality of life. Whilst the former values were generated using a UK- rather than Australian-based algorithm and may underestimate the HrQOL of Australians [63], the latter values were estimated using the 3L version which may be less sensitive and responsive to problems than the 5L version [7–10].

## Conclusions

The findings from this study provide the first population norms for South Australia based on a large, community-based sample by incorporating the newly developed UK general population scoring algorithm specifically developed for the five level version of the EQ-5D instrument [12]. The population-based values will facilitate empirical comparisons of the HrQOL of the general population with more specific patient groups and will be useful for estimating QALYs in economic evaluations in the Australian context. Overall, findings are consistent with EQ-5D-5L utility and VAS scores reported for other countries and indicate that the majority of South Australian adults report themselves in full health, with higher health status associated independently with younger age, male gender, longer duration of education, higher annual household income, employment and marriage or de facto relationships. Further research is recommended to update these population norms by applying the Australian general population specific scoring algorithm, currently in development, once this become publically available.

## Abbreviations

CI: Confidence intervals
DCE: Discrete choice experiment
EQ-5D: the EuroQol 5 dimensions
GLM: Generalized linear model
HOS: Health Omnibus Survey
HrQOL: Health-related quality of life
IQR: Interquartile range
IRSAD: Index of relative social advantage and disadvantage
MAUI: Multi-attribute utility instrument
QALY: Quality-adjusted life year
SD: Standard deviation
STROBE: Strengthening the Reporting of Observational Studies in Epidemiology
TTO: Time trade off
EQ-VAS: Visual analogue scale

## Footnotes

1 https://researchdata.ands.org.au/developing-an-australian-life-instrument/455165.

## References

[1] Hjelmgren J, Berggren F, Andersson F (2001). Health economic guidelines--similarities, differences and some implications. Value Health.

[2] Sullivan SM, Wells G, Coyle D (2015). What guidance are economists given on How to present economic evaluations for policymakers? A systematic review. Value Health.

[3] Merlo G, Page K, Ratcliffe J, Halton K, Graves N (2015). Bridging the Gap: exploring the barriers to using economic evidence in healthcare decision making and strategies for improving uptake. Applied Health Economics and Health Policy.

[4] Drummond M, Sculpher M, Torrance G, O’Brien B, Stoddart G (2005). Methods for the economic evaluation of health care programmes.

[5] Brazier J, Ratcliffe J, Tsuchiya A, Salomon J (2016). Measuring and Valuing Health Benefits for Economic Evaluation.

[6] Herdman M, Gudex C, Lloyd A, Janssen M, Kind P, Parkin D, Bonsel G, Badia X (2011). Development and preliminary testing of the new five-level version of EQ-5D (EQ-5D-5L). Qual Life Res.

[7] Feng Y, Devlin N, Herdman M (2015). Assessing the health of the general population in England: how do the three- and five-level versions of EQ-5D compare?. Health and Quality of Life Outcomes.

[8] Agborsangaya CB, Lahtinen M, Cooke T, Johnson JA (2014). Comparing the EQ-5D 3L and 5L: measurement properties and association with chronic conditions and multimorbidity in the general population. Health Qual Life Outcomes.

[9] Craig BM, Pickard AS, Lubetkin EI (2014). Health problems are more common, but less severe when measured using newer EQ-5D versions. J Clin Epidemiol.

[10] Janssen MF, Pickard AS, Golicki D, Gudex C, Niewada M, Scalone L, Swinburn P, Busschbach J (2013). Measurement properties of the EQ-5D-5L compared to the EQ-5D-3L across eight patient groups: a multi-country study. Qual Life Res.

[11] Garcia-Gordillo MA, Adsuar JC, Olivares PR. Normative values of EQ-5D-5L: in a Spanish representative population sample from Spanish Health Survey, 2011. Qual Life Res. 2015. 10.1007/s11136-015-1164-7 26482825

[12] Devlin N, Shah K, Feng Y, Mulhern B, van Hout B (2016). Valuing health-related quality of life: an EQ-5D-5L value set for England.

[13] Ravens-Sieberer U (2002). Measuring and monitoring quality-of-life in population surveys: still a challenge for public health research. Soz Praventivmed.

[14] Kularatna S, Whitty JA, Johnson NW, Jayasinghe R, Scuffham PA (2014). EQ-5D-3L derived population norms for health related quality of life in Sri Lanka. PLoS One.

[15] Banham D, Hawthorne G, Goldney R, Ratcliffe J (2014). Health-related quality of life (HRQoL) changes in South Australia: comparison of burden of disease morbidity and survey-based health utility estimates. Health Qual Life Outcomes.

[16] Health Quality Council of Alberta (2014). 2014 Alberta population norms for EQ-5D-5L.

[17] Hinz A, Kohlmann T, Stobel-Richter Y, Zenger M, Brahler E (2014). The quality of life questionnaire EQ-5D-5L: psychometric properties and normative values for the general German population. Qual Life Res.

[18] Scalone L, Cortesi PA, Ciampichini R, Cesana G, LG M (2015). Health related quality of life norm data of the Italian general population: results using the EQ-5D-3L and EQ-5D-5L instruments. Epidemiology, biostatistics and public health.

[19] Shiroiwa T, Fukuda T, Ikeda S, Igarashi A, Noto S, Saito S, Shimozuma K (2015). Japanese population norms for preference-based measures: EQ-5D-3L, EQ-5D-5L, and SF-6D. Qual Life Res.

[20] Golicki D, Niewada M. EQ-5D-5L Polish population norms. Arch Med Sci 2015:1–10. 10.5114/aoms.2015.52126 PMC5206353 28144271

[21] Augustovski F, Rey-Ares L, Irazola V, Garay OU, Gianneo O, Fernandez G, Morales M, Gibbons L, Ramos-Goni JM (2016). An EQ-5D-5L value set based on Uruguayan population preferences. Qual Life Res.

[22] Clemens S, Begum N, Harper C, Whitty J, Scuffham P (2014). A comparison of EQ-5D-3L population norms in Queensland, Australia, estimated using utility value sets from Australia, the UK and USA. Qual Life Res.

[23] Konig HH, Bernert S, Angermeyer MC, Matschinger H, Martinez M, Vilagut G, Haro JM, de Girolamo G, de Graaf R, Kovess V, Alonso J (2009). Comparison of population health status in six european countries: results of a representative survey using the EQ-5D questionnaire. Med Care.

[24] Johnson JA, Pickard AS (2000). Comparison of the EQ-5D and SF-12 health surveys in a general population survey in Alberta, Canada. Med Care.

[25] Sun S, Chen J, Johannesson M, Kind P, Xu L, Zhang Y, Burström K (2011). Population health status in China: EQ-5D results, by age, sex and socio-economic status, from the National Health Services Survey 2008. Qual Life Res.

[26] Sorensen J, Davidsen M, Gudex C, Pedersen KM, Bronnum-Hansen H (2009). Danish EQ-5D population norms. Scand J Public Health.

[27] Saarni SI, Harkanen T, Sintonen H, Suvisaari J, Koskinen S, Aromaa A, Lonnqvist J (2006). The impact of 29 chronic conditions on health-related quality of life: a general population survey in Finland using 15D and EQ-5D. Qual Life Res.

[28] Golicki D, Niewada M (2015). General population reference values for 3-level EQ-5D (EQ-5D-3L) questionnaire in Poland. Pol Arch Med Wewn.

[29] Ferreira LN, Ferreira PL, Pereira LN, Oppe M (2014). EQ-5D Portuguese population norms. Qual Life Res.

[30] Abdin E, Subramaniam M, Vaingankar J, Luo N, Chong S (2015). Population norms for the EQ-5D index scores using Singapore preference weights. Qual Life Res.

[31] Burström K, Sun S, Gerdtham U-G, Henriksson M, Johannesson M, Levin L-Å, Zethraeus N (2014). Swedish experience-based value sets for EQ-5D health states. Qual Life Res.

[32] Perneger TV, Combescure C, Courvoisier DS (2010). General population reference values for the French version of the EuroQol EQ-5D health utility instrument. Value Health.

[33] Kind P, Dolan P, Gudex C, Williams A (1998). Variations in population health status: results from a United Kingdom national questionnaire survey. BMJ.

[34] Luo N, Johnson JA, Shaw JW, Feeny D, Coons SJ (2005). Self-reported health status of the general adult U.S. population as assessed by the EQ-5D and Health Utilities Index. Med Care.

[35] Kontodimopoulos N, Pappa E, Niakas D, Yfantopoulos J, Dimitrakaki C, Tountas Y (2008). Validity of the EuroQoL (EQ-5D) Instrument in a Greek General Population. Value Health.

[36] Billa G, Thakkar K, Jaiswar S, Dhodi D (2014). A cross-sectional study to evaluate the awareness and attitudes of physicians towards reducing the cost of prescription drugs, Mumbai. Applied Health Economics and Health Policy.

[37] Phillips J, Dal Grande E, Ritchie C, Abernethy AP, Currow DC (2015). A population-based cross-sectional study that defined normative population data for the life-space mobility assessment-composite score. J Pain Symptom Manag.

[38] Currow DC, Burns C, Agar M, Phillips J, McCaffrey N, Abernethy AP (2011). Palliative caregivers Who would Not take on the caring role again. J Pain Symptom Manag.

[39] Oppe M, Devlin NJ, van Hout B, Krabbe PFM, de Charro F (2014). A program of methodological research to arrive at the New international EQ-5D-5L valuation protocol. Value Health.

[40] StataCorp (2013). Stata statistical software: release 13. StataCorp LP.

[41] Wilson D, Wakefield M, Taylor A (1992). The South Australian health omnibus survey. Health Promotion Journal of Australia.

[42] Sun S, Chen J, Kind P, Xu L, Zhang Y, Burström K (2015). Experience-based VAS values for EQ-5D-3L health states in a national general population health survey in China. Qual Life Res.

[43] Szende A, Williams A (2004). Measuring Self-Reported Population Health: An International Perspective based on EQ-5D.

[44] Hawthorne G, Korn S, Richardson J (2013). Population norms for the AQoL derived from the 2007 Australian National Survey of Mental Health and Wellbeing. Aust N Z J Public Health.

[45] Hawthorne G, Osborne R (2005). Population norms and meaningful differences for the Assessment of Quality of Life (AQoL) measure. Aust N Z J Public Health.

[46] Norman R, Church J, van den Berg B, Goodall S (2013). Australian health-related quality of life population norms derived from the SF-6D. Aust N Z J Public Health.

[47] Cohen J, Cohen J (1988). The Significance of a product moment rs. Statistical power analysis for the behavioural sciences.

[48] Curtin F, Schulz P (1998). Multiple correlations and bonferroni’s correction. Biol Psychiatry.

[49] Altman D (1991). Practical statistics for medical research.

[50] Bland JM, Altman DG (1995). Multiple significance tests: the Bonferroni method. BMJ.

[51] Willan A, Briggs AH (2006). Statistical analysis of cost-effectiveness data.

[52] Manning WG, Mullahy J (2001). Estimating log models: to transform or not to transform?. J Health Econ.

[53] Glick H, Doshi J, Sonnad S, Polsky D (2015). Economic evaluation in clinical trials.

[54] Mihaylova B, Briggs A, O’Hagan A, Thompson SG (2011). Review of statistical methods for analysing healthcare resources and costs. Health Econ.

[55] Parkin D, Rice N, Devlin N (2010). Statistical analysis of EQ-5D profiles: Does the use of value sets bias inference?. Med Decis Mak.

[56] Feng Y, Parkin D, Devlin NJ (2014). Assessing the performance of the EQ-VAS in the NHS PROMs programme. Qual Life Res.

[57] Steptoe A, Deaton A, Stone AA (2015). Subjective wellbeing, health, and ageing. Lancet.

[58] Parkin D, Devlin N, Feng Y. What determines the shape of an EQ-5D index distribution? Med Decis Making. 2016. 10.1177/0272989X16645581 27112934

[59] Canadian Agency for Drugs and Technologies in Health: Guidelines for the economic evaluation of health technologies: Canada. 3rd edn. Ottawa; 2006.

[60] Pharmaceutical Benefits Advisory Committee (2013). Guidelines for preparing submissions to the Pharmaceutical Benefits Advisory Committee (Version 4.4).

[61] National Institute for Health and Care Excellence (2013). Guide to the methods of technology appraisal 2013.

[62] Council HP, Council HP (2013). State of Our Health: Health Status and Health Determinants of South Australians Working Draft for Discussion.

[63] Brennan DS, Teusner DN (2015). Comparing UK, USA and Australian values for EQ-5D as a health utility measure of oral health. Community Dent Health.

[64] National Institute for Clinical Excellence (2008). Guide to the methods of technology appraisal.

[65] Pullenayegum EM, Perampaladas K, Gaebel K, Doble B, Xie F (2015). Between-country heterogeneity in EQ-5D-3L scoring algorithms: how much is due to differences in health state selection?. Eur J Health Econ.

[66] Norman R, Cronin P, Viney R (2013). A pilot discrete choice experiment to explore preferences for EQ-5D-5L health states. Appl Health Econ Health Policy.

